# Niclosamide suppresses osteoclastogenesis and bone resorption: involvement of Signal Transducer And Activator Of Transcription 3 signaling

**DOI:** 10.1093/jbmrpl/ziag169

**Published:** 2026-10-19

**Authors:** Yikun Zhu, Huihao Zheng, Xijian Hu, Jing Chai, Xintian Liu

**Affiliations:** The Second Hospital of Shanxi Medical University, Endocrinology Department, Taiyuan, 030001, China; The Second Hospital of Shanxi Medical University, Endocrinology Department, Taiyuan, 030001, China; Key Laboratory of Bone and Soft Tissue Repair, The Second Hospital of Shanxi Medical University, Taiyuan, 030001, China; The Second Hospital of Shanxi Medical University, Endocrinology Department, Taiyuan, 030001, China; The Second Hospital of Shanxi Medical University, Endocrinology Department, Taiyuan, 030001, China

**Keywords:** niclosamide, osteoclast, osteoporosis, pyroptosis, STAT3

## Abstract

Niclosamide, known for its anticancer and anti-inflammatory effects via modulation of NF-κB and STAT3 signaling, has now been shown to suppress osteoclast differentiation and function through STAT3 inhibition. In vitro experiments demonstrated that niclosamide downregulates STAT3 activation, leading to reduced expression of genes involved in osteoclast differentiation, acid secretion, chemotaxis, and pyroptosis, thereby impairing multiple osteoclast biological behaviors. In vivo, niclosamide treatment attenuated bone loss in an ovariectomy-induced mouse model of osteoporosis (OP) by inhibiting osteoclast activity. The STAT3 activator colivelin partially reversed these niclosamide-induced effects on bone metabolism. Collectively, niclosamide inhibits both osteoclast formation and the bone-resorptive function of mature osteoclasts via suppression of the STAT3 pathway, suggesting a novel therapeutic approach for OP.

## Introduction

Bone undergoes continuous remodeling and renewal through the coupled actions of osteoclast-mediated bone resorption and osteoblast-mediated bone formation. The balance and homeostasis between these 2 cell types are essential for maintaining skeletal integrity, mechanical strength, and mineral homeostasis.^1^^,^^2^ Excessive osteoclast activity and increased bone resorption disrupt this remodeling equilibrium, leading to osteolytic disorders, such as osteoporosis (OP).^3^^,^^4^

The differentiation and maturation of osteoclasts are regulated by multiple cytokines. Multinucleated mature osteoclasts are formed through the fusion of osteoclast precursors (OCPs). Macrophage colony-stimulating factor (M-CSF) and RANKL serve as critical factors involved in osteoclast differentiation. Macrophage colony-stimulating factor is primarily secreted by osteoblasts and BM stromal cells and promotes the differentiation and proliferation of OCP cells by binding to its receptor on OCPs.^5^ RANKL, which is produced mainly by osteoblasts, mesenchymal stem cells, and T cells, binds to its receptor, RANK, and activates TNF receptor-associated factor 6 (TRAF6), subsequently leading to the activation of NF-κB and nuclear factor of activated T cells 1 (NFATc1), thereby driving osteoclast differentiation and fusion.^3^^,^^6–8^ Furthermore, during the process of bone resorption, mature osteoclasts attach their ruffled border to the bone surface and form an annular sealing zone around it. Proteins located on the ruffled border, such as the chloride–proton exchanger CLCN7 and the vacuolar-type H+-ATPase (V-ATPase), facilitate acidification of the resorption lacuna and dissolution of inorganic minerals. This acidic microenvironment enables key bone-resorbing enzymes, including cathepsin K (CTSK), to degrade the bone matrix, thereby executing the resorptive function of osteoclasts.^9–11^

The chemotactic migration of osteoclasts on bone surfaces is closely associated with C-X-C motif chemokine 12 (CXCL12) and sphingosine-1-phosphate (S1P). CXCL12, a survival factor for stem and progenitor cells, is involved in regulating their homing, chemotaxis, and directed differentiation.^12^^,^^13^ Sphingosine-1-phosphate is a biologically active natural sphingolipid that participates in the regulation of cell migration, differentiation, proliferation, and survival. Osteoclast precursors express 2 types of receptors, sphingosine-1-phosphate receptor 1 (S1PR1) and sphingosine-1-phosphate receptor 2 (S1PR2), which mediate chemoattraction and chemorepulsion, respectively. Sphingosine-1-phosphate receptor 1 recruits OCPs from the BM into peripheral blood, whereas S1PR2 guides OCPs from the circulation back to the BM.^14–18^ However, the potency of S1P is lower than that of CXCL12. Under physiological conditions, the BM contains high concentrations of CXCL12 and low concentrations of S1P. High levels of CXCL12 bind to C-X-C chemokine receptor type 4 (CXCR4), and low levels of S1P bind to S1PR2, collectively retaining OCPs within the BM. Conversely, when CXCL12 levels decrease in the BM, leading to reduced binding to CXCR4, and S1P levels increase, promoting binding to S1PR1, OCP recruitment into the bloodstream increases.^19^^,^^20^ Additionally, osteoblasts also express S1P receptors. Elevated S1P levels in the BM bind to these receptors and upregulate RANKL expression and downregulate osteoprotegerin expression in osteoblasts, thereby promoting osteoclastogenesis.^15^^,^^21^

Pyroptosis is a form of programmed cell death characterized by cellular swelling, plasma membrane rupture, the release of cellular contents, and the activation of inflammatory responses.^22^ In its initial phase, the NLRP3 inflammasome is activated and subsequently cleaves pro-caspase-1 into its active form, cleaved caspase-1. The activated caspase-1 then processes pro-IL-1β and pro-IL-18 into their mature forms and cleaves gasdermin D (GSDMD). The N-terminal fragment of GSDMD oligomerizes and forms transmembrane pores in the plasma membrane, leading to osmotic swelling, membrane rupture, and the release of intracellular mediators. This process triggers a strong inflammatory response^23^^,^^24^ and ultimately results in cell swelling, lysis, and death.^25^

Niclosamide can modulate cellular metabolism by influencing mitochondrial and lysosomal functions, as well as the activities of various enzymes, such as endonucleases and lactate dehydrogenase, thereby affecting the oxidative phosphorylation of proteins.^26^^,^^27^ Recent studies have demonstrated that niclosamide exerts pleiotropic effects and shows significant efficacy against colorectal cancer, prostate cancer, breast cancer, and acute myeloid leukemia by inhibiting signaling pathways, such as the Wnt/β-catenin and STAT3 pathways.^28–32^ STAT3 signaling is involved in bone remodeling and the pathogenesis of OP. STAT3 promotes the differentiation and proliferation of osteoclasts and increases bone resorption by regulating the transcription of the key transcription factor NFATc1, thereby driving the expression of osteoclast-specific genes.^33^ Conversely, the Wnt/β-catenin signaling pathway plays a crucial regulatory role in the osteogenic differentiation of BM mesenchymal stem cells and bone formation. Dysregulation of this pathway is closely associated with the development of various bone metabolic diseases.^34^ Whether the inhibitory effects of niclosamide on osteoclasts can be reversed by activating STAT3 warrants further investigation.

In this study, the effects of niclosamide on the development and maturation of primary mouse osteoclasts were investigated, and the underlying molecular mechanisms were explored. The therapeutic potential of niclosamide was further validated in an ovariectomy-induced OP mouse model. Additionally, the STAT3 activator colivelin was used to determine whether the inhibitory effects of niclosamide could be reversed.

## Materials and methods

### Mice and reagents

Female C57BL/6 mice aged 4-8 wk were obtained from the SPF-grade Laboratory Animal Center of Shanxi Medical University (Production License No. SCXK [Jin] 2024-0004) to ensure standardized and reliable experimental animal sources. All animal procedures were approved by the Medical Ethics Committee of the Second Hospital of Shanxi Medical University (Approval No. DW2025007) and were conducted in strict accordance with ethical guidelines for animal research.

FBS, MEM alpha, RANKL, M-CSF, penicillin–streptomycin, L-glutamine, sterile PBS, red blood cell lysis buffer, and tartrate-resistant acid phosphatase (TRAP) staining kits were purchased from Amizona Scientific. Niclosamide, colivelin, and acridine orange staining reagents were obtained from MedChemExpress. Antibodies against NFATc1, CTSK, microphthalmia-associated transcription factor (MITF), B lymphocyte-induced maturation protein-1 (Blimp-1), B-cell lymphoma 6 (Bcl-6), sphingosine kinase 1 (SPHK1), S1PR1, S1PR2, SPNS lysolipid transporter 1 (SPNS2), CXCL12, CXCR4, apoptosis-associated speck-like protein containing a CARD (ASC), GSDMD, interleukin-1β (IL-1β), NLRP3, and glyceraldehyde-3-phosphate dehydrogenase (GAPDH) were obtained from Abcam. Antibodies targeting cleaved caspase-1, V-ATPase, and CLCN7 were procured from Thermo Fisher Scientific.

### Bone marrow macrophage (BMM) isolation

MEM alpha supplemented with FBS was prepared. Mice aged 6-8 wk were euthanized by cervical dislocation. Both tibiae and femora were aseptically harvested from each mouse. Using sterile scissors and forceps, the bones were transferred to a culture dish containing PBS. The bone ends were carefully removed with scissors, and the BM was flushed into the dish using a 5 mL syringe filled with PBS. The resulting marrow suspension was filtered through a cell strainer. The cell suspension was collected and centrifuged at 350 g for 5 min. After the supernatant was discarded, the pellet was resuspended in 10 mL of complete medium to obtain mouse BM cells. The cell suspension was then seeded into 10 cm culture dishes and incubated overnight at 37 °C in a humidified atmosphere containing 7% CO₂.

### Osteoclast differentiation

The isolated BMMs were centrifuged at 350 g for 5 min, and the cell pellet was collected after the supernatant was removed. Next, 500 μL of red blood cell lysis buffer (Amizona Scientific) was added to resuspend the cells, followed by an incubation at room temperature for 90 s. Subsequently, 10 mL of PBS was added, and the mixture was centrifuged again at 350 g for 5 min. After the supernatant was discarded, the cell pellet was resuspended in 10 mL of complete medium, and the cell concentration was adjusted to 1.0 × 10^5^ cells/mL using complete medium. The adjusted cell suspension was then seeded into culture plates at the following volumes: 3 mL for 6-well plates, 1 mL for 24-well plates, and 100 μL for 96-well plates. M-CSF was added to each well at a final concentration of 40 ng/mL, and the cells were incubated overnight at 37 °C with 7% CO₂. The following day, the cells were supplemented with 40 ng/mL RANKL, and the culture was continued for 3 d. After 3 d, the medium was replaced with fresh complete medium containing both RANKL and M-CSF, and the incubation was prolonged for an additional 2 d. Mature multinucleated osteoclasts could be observed by the end of this culture period.

### Groups and interventions

Based on the types and concentrations of the intervention agents, the cells were divided into 3 groups: the DMSO control group, the niclosamide (1.5 μM) group, and the niclosamide (1.5 μM) + colivelin (2 μM) rescue group. Treatments were applied at two key time points to determine the specific stage of osteoclast differentiation at which niclosamide acts: the early phase (day 0, upon initial RANKL stimulation) and the late phase (day 3, during medium replacement with fresh RANKL-containing medium) (Figure 1A). Additionally, primary osteoblasts were co-cultured with osteoclasts under the same experimental conditions to assess whether the presence of osteoblasts influences the observed outcomes.

**Figure 1:**
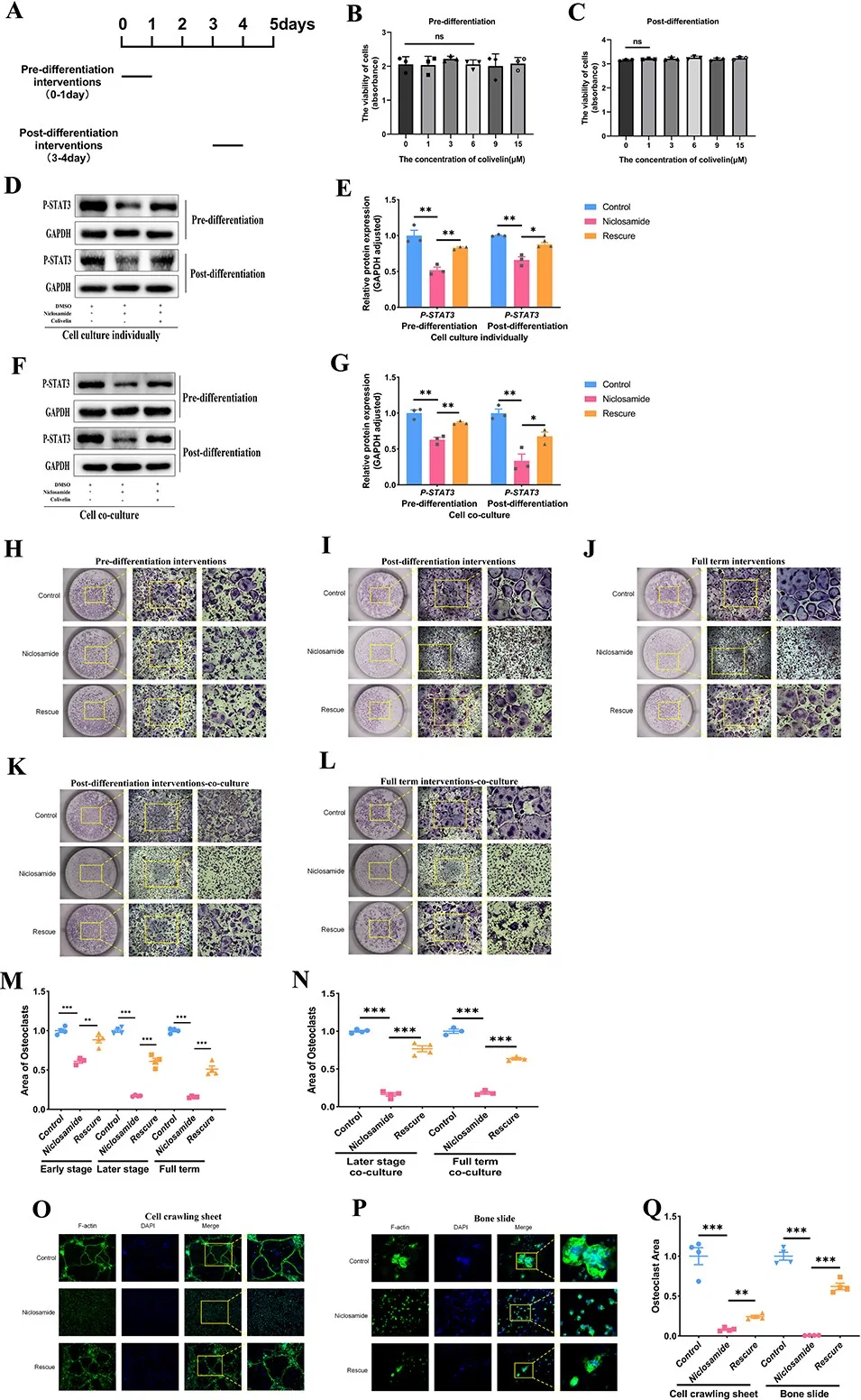
(A) Schematic of the time points at which the drug was administered. (B) Dose-dependent effect of colivelin on osteoclast viability when it was administered during early differentiation. (C) Dose-dependent effect of colivelin on osteoclast viability when it was administered during late differentiation. (D) p-STAT3 levels in an osteoclast monoculture after the drug intervention. (F) p-STAT3 levels in osteoclast–osteoblast cocultures after the drug intervention. (E and G) Quantification of p-STAT3 levels in (D) and (F), respectively. (H) TRAP staining of osteoclasts in monoculture after early-stage intervention (scale bars: 400, 100, and 50 μm). (I) TRAP staining of osteoclasts in monoculture after late-stage intervention. (J) TRAP staining of osteoclasts in monoculture after continuous intervention throughout differentiation. (K) TRAP staining of osteoclasts in coculture after late-stage intervention. (L) TRAP staining of osteoclasts in coculture after continuous intervention. (M) Quantification of the mature osteoclast area in (H)-(J). (N) Quantification of the mature osteoclast area in (K) and (L). (O and P) Phalloidin and DAPI staining of osteoclasts cultured on glass coverslips (scale bars: 100 and 50 μm) and bone slices (scale bars: 100 and 33 μm) after late-stage intervention. (Q) Quantification of the mature osteoclast area in (O) and (P) (^**^p < .001, ^**^p < .01, and ^*^p < .05).

### C‌CK-8 cell viability assay

The cytotoxicity of the STAT3 activator colivelin was evaluated using a CCK-8 assay. Cells were treated with different concentrations of colivelin (1, 3, 6, 9, and 15 μM) or DMSO (control) at 2 time points: upon initial RANKL addition (day 0) and during medium replacement with fresh RANKL-containing medium (day 3). Twenty-four hours after each treatment, 10 μL of CCK-8 solution (Dojindo Molecular Technologies) was added to each well, and the plates were further incubated for 2 h with 7% CO₂. The absorbance at 450 nm was measured using a microplate reader to quantify cell viability and proliferation.

### TRAP staining

Following the removal of the culture medium, the cells were gently washed with sterile PBS once and fixed with the fixation solution for 30 s at room temperature, after which they were thoroughly rinsed with PBS. For the preparation of the staining solution for each well of a 24-well plate, 2.5 μL of sodium nitrite solution and 2.5 μL of fast garnet GBC base solution were first mixed and incubated at room temperature for 2 min. Separately, 18 μL of a mixture containing acetate solution, naphthol AS-BI phosphoric acid solution, and tartrate solution was added to 230 μL of double-distilled water, and the 2 solutions were then combined and mixed thoroughly. After the PBS was removed from the wells, 250 μL of the prepared staining solution was added to each well, followed by an incubation at 37 °C for 1 h. Stained cells were subsequently observed under an optical microscope.

### F-actin ring staining

Cells were fixed with 4% paraformaldehyde for 20 min, washed with PBS, and permeabilized with 0.5% Triton X-100 at room temperature for 10 min. After washes with PBS, the cells were blocked with 5% FBS for 30 min at room temperature. The fixed cells were then incubated with diluted phalloidin (MedChemExpress) in the dark at room temperature for 30 min, followed by washes with PBS. Nuclei were counterstained with 4′,6-diamidino-2-phenylindole (DAPI; Thermo Fisher Scientific) for 5 min in the dark. Finally, the samples were observed under a confocal microscope.

### Immunofluorescence staining

Bone marrow macrophages were seeded on glass coverslips and treated with the appropriate compounds for 24 h at either the early or late stage of differentiation. Following treatment, the cells on the coverslips were washed with PBS, fixed with 4% paraformaldehyde, permeabilized with 0.1% Triton X-100, and blocked with 5% bovine serum albumin (BSA). The cells were subsequently incubated overnight at 4 °C with specific monoclonal antibodies against NFATc1 and CTSK (diluted 1:50). The next day, after washes with PBS, the cells were incubated for 2 h at room temperature with a FITC-conjugated goat anti-mouse IgG secondary antibody (Abcam). After the immunostaining procedure was complete, the nuclei were counterstained with DAPI. Fluorescence images were acquired using a fluorescence microscope.

### Immunohistochemical staining

Mouse femoral sections were deparaffinized in xylene and rehydrated through a graded ethanol series, followed by antigen retrieval using enzymatic digestion. After the tissue sections were washed with PBS, a hydrophobic barrier pen was used to outline them. The sections were then incubated with a blocking solution at room temperature for 1 h in a humidified chamber, washed with PBS, and subsequently incubated overnight at 4 °C with a primary antibody against NFATc1 (1:100 dilution). The following day, the sections were washed with PBS and incubated with a horseradish peroxidase (HRP)-conjugated goat anti-rabbit IgG secondary antibody for 30 min at room temperature. After additional PBS washes, color development was performed using a 3,3′-diaminobenzidine substrate, and the nuclei were counterstained with hematoxylin. Finally, the sections were scanned using a whole-slide digital scanner for the pathological analysis.

### Acridine orange staining

After the osteoclasts in the DMSO control group had fully differentiated, the culture medium was removed from all the groups, and the cells were gently washed once with sterile PBS. Acridine orange staining reagent (1 μM) was subsequently added to stain the cells for 20 min at 37 °C. Then, the staining solution was discarded, and the cells were rinsed again with PBS. The staining patterns of osteoclasts in each group were immediately examined under a fluorescence microscope.

### Bone resorption assay

Bone marrow macrophages were seeded onto bovine cortical bone slices in 24-well plates at a density of 1 × 10^5^ cells per well. Cells were then cultured for 9 d with the respective interventions to promote osteoclast differentiation and bone resorption. Following the culture period, bone slices were harvested and thoroughly rinsed with PBS to remove adherent cells. After fixation, the slices were imaged using a SEM. Resorption pit areas on the slices were quantitatively analyzed.

### ELISA

In vitro, culture supernatants from cells were collected 24 h after the drug intervention at the indicated differentiation stages. In vivo, blood was collected immediately via cardiac puncture following euthanasia, transferred to 1.5 mL tubes, and allowed to clot at room temperature for 30 min. After clot removal by centrifugation at 5500 g for 15 min in a refrigerated centrifuge, the resulting serum was aliquoted and stored at −80 °C. S1P concentrations in both the culture supernatant and serum samples were measured using a commercial ELISA kit according to the manufacturer’s instructions.

### Total RNA extraction and quantitative real-time RT-PCR

Total RNA was extracted from osteoclasts using the miRNeasy Mini Kit (Qiagen) and reverse-transcribed into cDNA with PrimeScript RT Master Mix (Takara). Real-time PCR was subsequently performed using SYBR Premix Ex Taq (Takara) along with gene-specific primers. The sequences of all the primers used are listed in the supplementary data (Table 1).

### Western blot analysis

Total protein was extracted from cells using RIPA lysis buffer. Protein samples were separated on 12% SDS-PAGE gels and transferred onto PVDF membranes. After blocking at room temperature for 1 h, the membranes were incubated overnight with specific primary antibodies, followed by an incubation with HRP-conjugated anti-mouse or anti-rabbit IgG secondary antibodies. The membranes were then treated with enhanced chemiluminescence substrate and imaged using a digital imaging system. Protein band intensities were quantified with ImageJ software (National Institutes of Health) by measuring the integrated optical density of each band.

### In vivo experiments

Sexually mature female C57BL/6 mice aged 8-12 wk were used in this study. Sham-operated mice underwent laparotomy and exposure of the ovaries without ovariectomy. Prior to surgery, all the mice were fasted for 12 h. After thorough anesthesia was established with inhaled anesthetic, the surgical site was shaved and disinfected with iodine. Under aseptic conditions, a 1-2 cm longitudinal incision was made in the lateral abdomen, followed by layer-by-layer dissection of the skin, fascia, and adipose tissue. The ovary, which appeared as a pink mulberry-like structure embedded in fat, was identified, exteriorized together with the oviduct, ligated together with the surrounding fat, and excised. The stump was returned to the abdominal cavity, and the incision was rinsed with sterile saline before being sutured and disinfected. The contralateral ovary was removed using the same procedure. No intraoperative mortality occurred. Postoperatively, all the mice were allowed free access to food and water. The niclosamide group received daily oral niclosamide (30 mg/kg). The colivelin group received oral niclosamide (30 mg/kg) combined with an intraperitoneal injection of colivelin (1 mg/kg) daily. After 8 wk, the mice were euthanized, and the left femur of each animal was analyzed using high-resolution micro-CT. The micro-CT experimental parameters and corresponding reports have been uploaded to the supplementary file.

### Statistical analyses

The data are presented as the means ± SDs. We statistically analyzed the differences among groups using ANOVA with the Statistical Package for the Social Sciences software (SPSS, version 26.0), and *p* < .05 was considered to indicate statistical significance.

## Results

### Effects of colivelin on the viability of primary mouse BMMs

The results of the cytotoxicity assay showed that treatment with 0-15 μM colivelin did not affect cell viability. Based on previous findings from our research group, niclosamide at concentrations less than 1.75 μM also exhibited no significant toxicity toward OCPs.^35^ Accordingly, niclosamide (1.5 μM) and colivelin (2 μM) were selected as experimental interventions in this study (Figure 1B and C).

### Inhibitory effect of Niclosamide on STAT3

Cells were treated with 1.5 μM niclosamide and/or 2 μM colivelin at either the early or late stage of osteoclast differentiation under both monoculture and osteoblast–osteoclast coculture conditions. The results demonstrated that niclosamide, a STAT3 inhibitor, significantly reduced the level of phosphorylated STAT3 (p-STAT3). This inhibitory effect was effectively reversed by the STAT3 activator colivelin (Figure 1D-G).

### Effects of niclosamide on osteoclast differentiation and maturation

Two culture systems were used, primary osteoclast monoculture and coculture with primary osteoblasts, to determine whether niclosamide affects the differentiation and maturation of primary mouse osteoclasts. The drugs were administered at different stages of differentiation. Furthermore, primary mouse cells were seeded on glass coverslips and bone slices were stained to detect F-actin and assess mature osteoclast formation. The drug intervention suppressed osteoclast fusion and differentiation, and these inhibitory effects were reversed by the STAT3 activator colivelin (Figure 1O-Q).

### Effects of niclosamide on the expression of genes related to osteoclast differentiation

RT-PCR, Western blotting, and immunofluorescence staining were performed to analyze changes in gene expression during the RANKL-induced differentiation of mouse BMMs into osteoclasts. The results indicated that treatment with 1.5 μM niclosamide significantly downregulated the expression of NFATc1, MITF, Blimp-1, BCL-6, and TRAP5, whereas cotreatment with colivelin upregulated their expression (Figure 2). These findings suggest that niclosamide may inhibit RANKL-induced osteoclast differentiation by suppressing the expression of the transcription factors NFATc1 and MITF and increasing the expression of proteins in the Blimp-1–BCL-6 axis, which negatively regulates NFATc1 activity.

**Figure 2:**
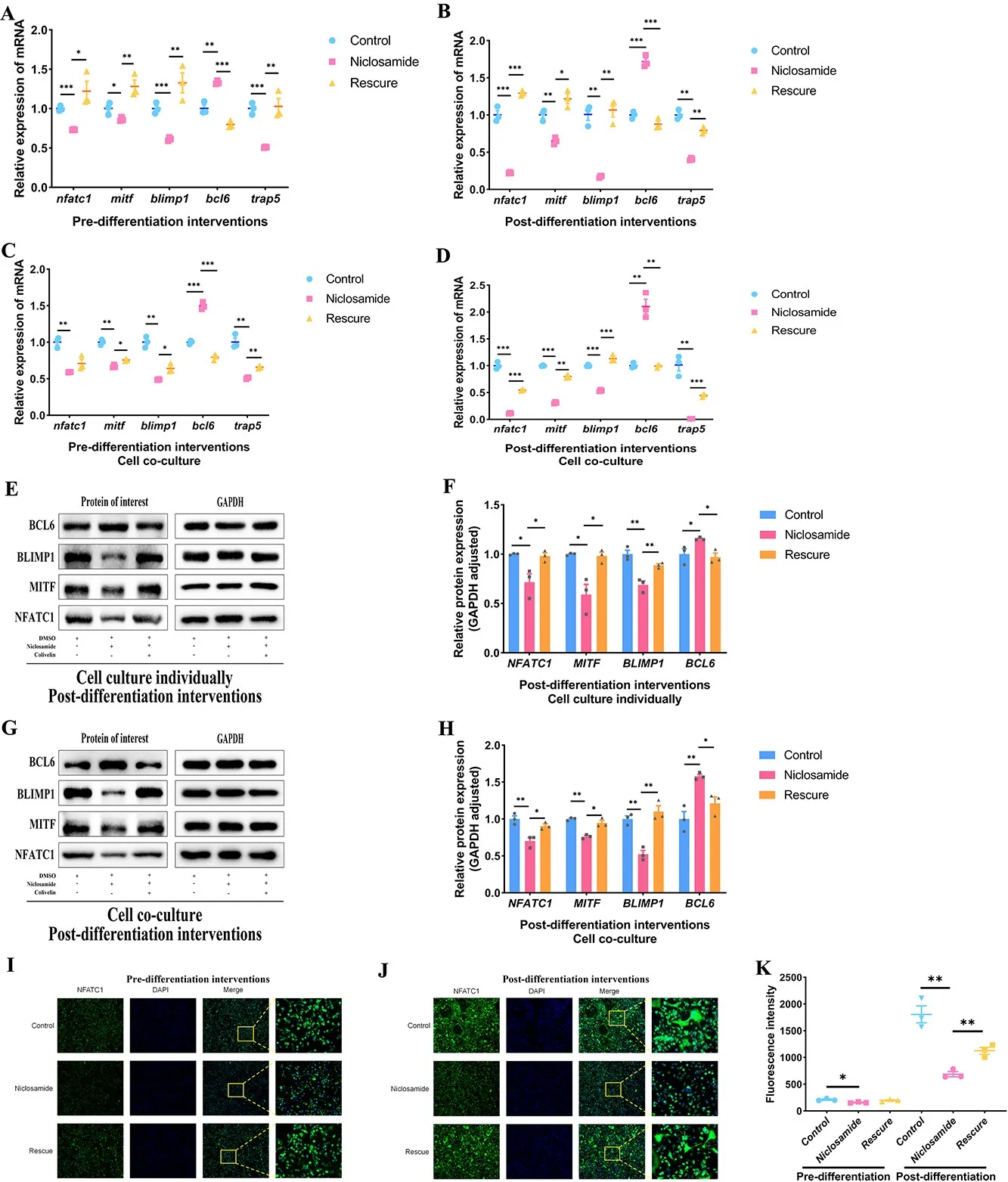
(A and B) Quantitative PCR quantification of osteoclast monocultures: (A) early-stage intervention and (B) late-stage intervention. (C and D) Quantitative PCR quantification of osteoclast–osteoblast cocultures: (C) early-stage intervention and (D) late-stage intervention. (E) Western blot analysis of osteoclast monocultures after late-stage intervention. (G) Western blot analysis of osteoclast–osteoblast cocultures after late-stage intervention. (F and H) Quantification of the Western blot results corresponding to (E) and (G), respectively. (I) Immunofluorescence staining for NFATc1 in osteoclast monocultures following intervention at the early differentiation stage. (J) Immunofluorescence staining for NFATc1 in osteoclast monocultures following intervention at the late differentiation stage (scale bars: 100 and 25 μm). (K) Quantification of the fluorescence intensity shown in (I) and (J) (^***^p < .001, ^**^p < .01, and ^*^p < .05).

### Effects of niclosamide on osteoclast function and related genes

We investigated the effect of niclosamide on osteoclast function. In vitro, acridine orange staining was performed to evaluate acid secretion by osteoclasts (Figure 3A-C). Bone resorption function was assessed by measuring the resorption pit area on bone slices using SEM (Figure 3I and J). We determined the effect of niclosamide on the acid-secreting function of osteoclasts by examining changes in the expression of genes associated with acid secretion, namely, V-ATPase, CLCN7, and the key bone resorption enzyme CTSK, in both osteoclast monoculture and coculture systems after treatment with 1.5 μM niclosamide. These genes were significantly downregulated (Figure 4F-M). Furthermore, drug intervention at different stages of osteoclast differentiation reduced CTSK expression, as shown by immunofluorescence staining. This suppression was reversed by cotreatment with colivelin (Figure 4A-C). These results indicate that niclosamide inhibits osteoclast-mediated bone resorption and that this inhibitory effect can be reversed by colivelin.

**Figure 3:**
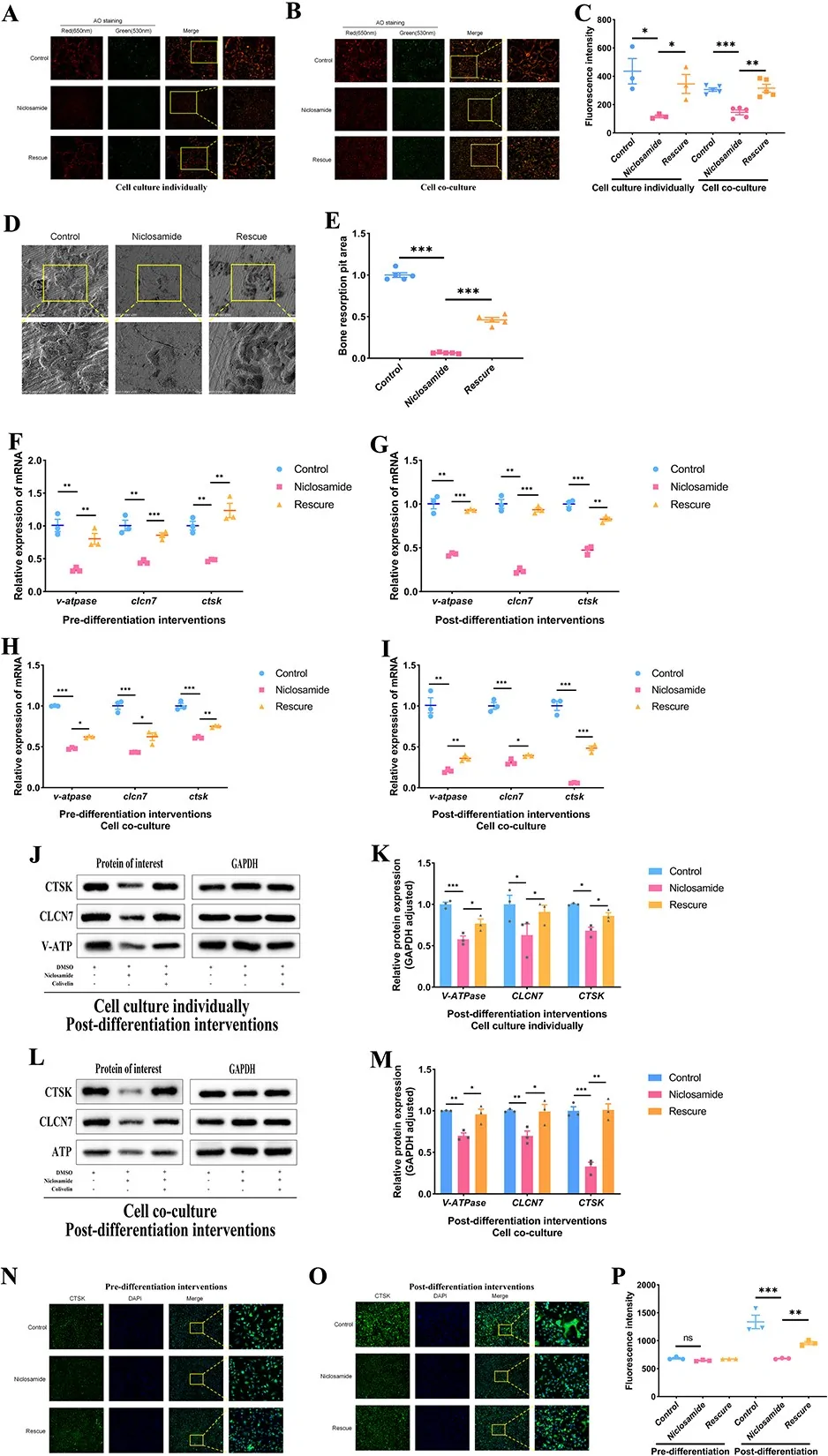
(A) Acridine orange staining of osteoclasts in monoculture. (B) Acridine orange staining of osteoclasts in coculture (scale bars: 100 and 50 μm). (C) Quantification of red fluorescence intensity in (A) and (B). (D) Resorption pits on bone slices (scale bars: 50 and 20 μm). (E) Quantification of the resorption pit area. (F and G) Quantitative PCR analysis of osteoclast monocultures: (F) early-stage intervention and (G) late-stage intervention. (H and I) Quantitative PCR analysis of osteoclast–osteoblast cocultures: (H) early-stage intervention and (I) late-stage intervention. (J) Western blot analysis of osteoclast monocultures after late-stage intervention. (L) Western blot analysis of osteoclast–osteoblast cocultures after late-stage intervention. (K and M) Quantification of the Western blot results corresponding to (J) and (L), respectively. (N) Immunofluorescence staining for CTSK in osteoclast monocultures after early-stage intervention. (O) Immunofluorescence staining for CTSK in osteoclast monocultures after late-stage intervention (scale bars: 100 and 25 μm). (P) Quantification of the fluorescence intensity in (N) and (O) (^***^p < .001, ^**^p < .01, and ^*^p < .05).

**Figure 4:**
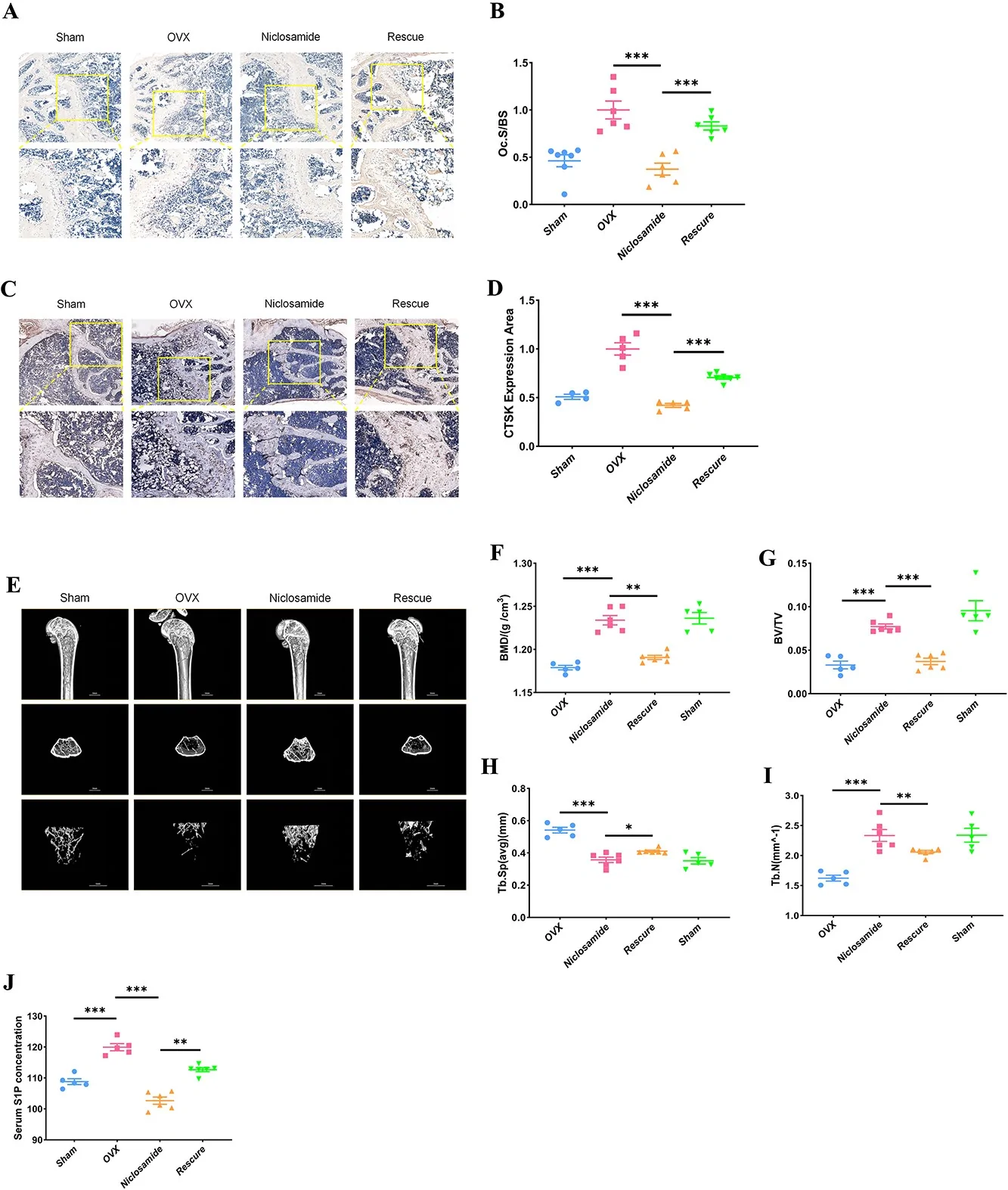
(A) Tartrate-resistant acid phosphatase staining of femoral sections from ovariectomized osteoporotic mice after drug treatment (scale bars: 200 and 100 μm). (B) Quantification of the osteoclast surface per bone surface (Oc.S/BS). (C) Immunohistochemical staining for CTSK in femoral sections from ovariectomized osteoporotic mice (scale bars: 200 and 100 μm). (D) Quantitative analysis of the immunohistochemical staining shown in (C). (E) Three-dimensional micro-CT reconstructions of femora from ovariectomized osteoporotic mice (*n* = 6, scale bar: 1 mm). (F-I) Quantitative analysis of the BMD, trabecular bone volume/total volume (BV/TV), trabecular separation (Tb.Sp), and trabecular number (Tb.N) in mouse femora. (J) Serum S1P concentration in ovariectomized osteoporotic mice (^***^p < .001, ^**^p < .01, and ^*^p < .05).

### Effects of niclosamide on OVX-induced bone loss

To investigate the in vivo effect of niclosamide, a mouse model of ovariectomy-induced OP was established. In this model, TRAP staining demonstrated that niclosamide inhibited osteoclast differentiation (Figure 4A and B). Consistently, immunohistochemical staining of femoral sections from these ovariectomized mice revealed decreased expression of the key bone resorption enzyme CTSK (Figure 4C and D). Micro-CT analysis of the femora was subsequently performed to obtain 3-dimensional reconstructions, and the BMD, trabecular bone volume/total volume (BV/TV), trabecular separation (Tb.Sp), and trabecular number (Tb.N) were quantified to evaluate bone loss (Figure 4E-I). Furthermore, serum concentrations of sphingosine-1-phosphate (S1P) were measured using ELISA (Figure 4J).

### Effects of niclosamide on osteoclast chemotaxis

Chemotaxis is essential for bone resorption by osteoclasts. We evaluated the effect of niclosamide on the expression of chemotaxis-related genes by first assessing the expression of S1P-associated genes and proteins, including the lipid kinase SPHK1, receptors S1PR1 and S1PR2, transporter SPNS2, and the chemokine CXCL12 and its receptor, using quantitative real-time PCR and Western blotting (Figure 5A-J). S1P concentrations in the culture supernatants of primary mouse BMMs at different differentiation stages were subsequently measured using ELISA (Figure 5K and L). The results showed that niclosamide suppressed the expression of key chemotaxis-related genes and reduced S1P levels. These inhibitory effects were significantly reversed by colivelin treatment.

**Figure 5:**
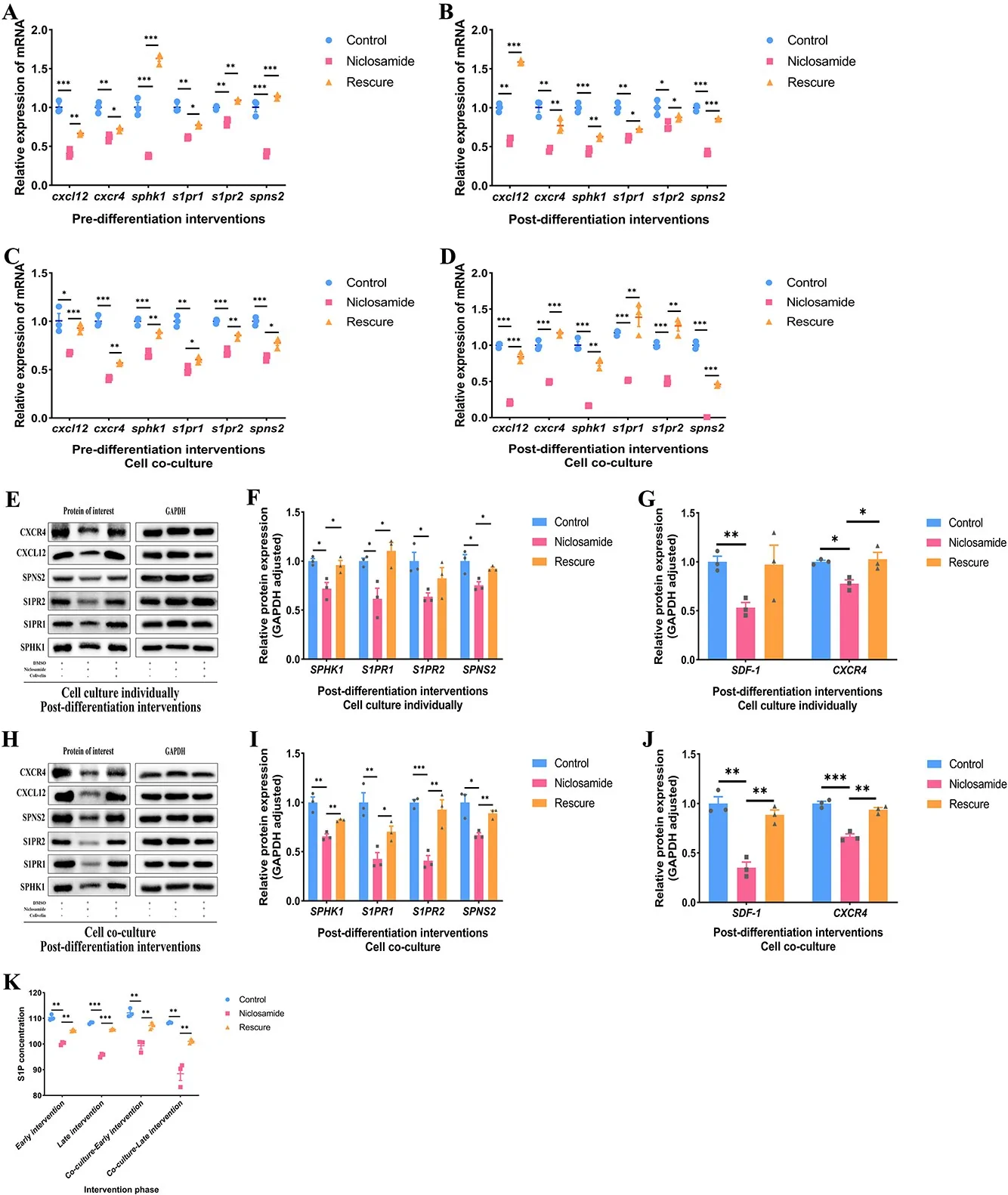
(A and B) Quantitative PCR analysis of osteoclast monocultures: (A) early-stage intervention and (B) late-stage intervention. (C and D) Quantitative PCR analysis of osteoclast–osteoblast cocultures: (C) early-stage intervention and (D) late-stage intervention. (E) Western blot analysis of osteoclast monocultures after late-stage intervention. (H) Western blot analysis of osteoclast–osteoblast cocultures after late-stage intervention. (F, G, I, and J) Quantification of the Western blot results corresponding to panels (E) and (H), respectively. (K) S1P concentration in culture supernatants of primary mouse BMMs at different differentiation stages (^***^p < .001, ^**^p < .01, and ^*^p < .05).

### Effects of niclosamide on osteoclast pyroptosis

As described above, we investigated the effects of niclosamide on osteoclast differentiation, function, and chemotaxis. Given the potential link between drug action and cell death, we further examined whether niclosamide influences osteoclast survival. After osteoclast differentiation and fusion, the cells were treated with niclosamide, and their viability was assessed at 0, 1, 3, 6, 12, and 24 h. Tartrate-resistant acid phosphatase staining performed at 24 h revealed an increased number of dead mature osteoclasts in the niclosamide-treated group (Figure 6A-D and Figure S1). Cell morphology was examined using SEM after 3 h of drug treatment to explore the underlying mechanism (Figure 6F). The observed morphological changes suggested a possible association with pyroptosis. The expression levels of pyroptosis-related genes, including NLRP3, ASC, and GSDMD, were subsequently measured (Figure 6G-I). These results indicated that niclosamide induced pyroptosis in mature osteoclasts, whereas colivelin did not alter pyroptosis.

**Figure 6:**
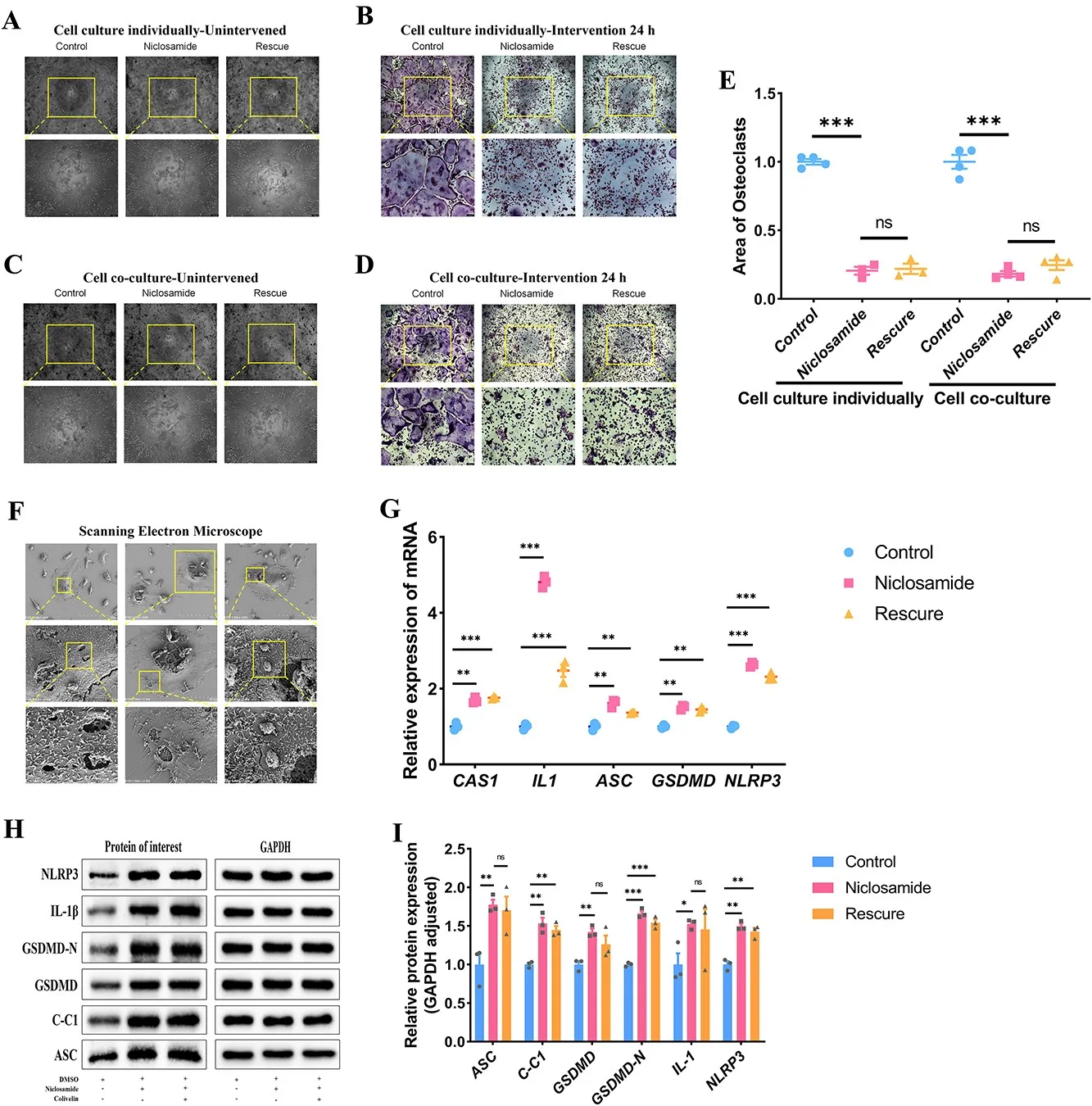
(A) Images of osteoclast monocultures captured immediately after the drug intervention following differentiation and fusion (0 h). (B) Tartrate-resistant acid phosphatase staining of osteoclast monocultures 24 h after the drug intervention following differentiation and fusion. (C) Images of osteoclast–osteoblast cocultures captured immediately after the drug intervention following differentiation and fusion (0 h). (D) Tartrate-resistant acid phosphatase staining of osteoclast–osteoblast cocultures at 24 h after the drug intervention following differentiation and fusion (scale bars: 100 and 50 μm). (E) Quantification of the osteoclast area in (A)-(D). (F) SEM images of mature osteoclasts captured 3 h after drug intervention. (G) Quantitative PCR analysis of osteoclast monocultures 24 h after the drug intervention following differentiation and fusion. (H) Western blot analysis of osteoclast monocultures 24 h after the drug intervention following differentiation and fusion. (I) Quantification of the Western blot results shown in (H) (^***^p < .001, ^**^p < .01, and ^*^p < .05).

## Discussion

Osteoporosis is characterized by an imbalance between osteoclast-mediated bone resorption and osteoblast-mediated bone formation, leading to a reduced bone mass, deteriorated bone microarchitecture, and compromised bone strength, which collectively increase the risk of low-trauma fractures. These fractures are major causes of disability and mortality in affected patients, making OP a condition of growing clinical and public health concern.^36^ Alterations in osteoclast differentiation and function play a central role in the pathogenesis, progression, and therapeutic targeting of OP.^3^^,^^37^ Therefore, research focusing on osteoclast-directed mechanisms may provide novel insights into this disease and inspire innovative therapeutic strategies.

Niclosamide targets multiple signaling pathways, including wingless-type MMTV integration site family (Wnt)/β-catenin, STAT3, NF-κB, Notch, and mammalian target of rapamycin complex 1 (mTOR). Recent studies have shown its potent anticancer effects on intestinal tumors, prostate cancer, breast cancer, and acute myeloid leukemia.^28–32^ These pathways also play important roles in bone homeostasis. For instance, in vivo experiments have shown that osteoclast-specific STAT3 KO leads to decreased NFATc1 transcription and results in increased bone mass in mice.^33^ Our data indicate that niclosamide, a STAT3 inhibitor, significantly suppresses various biological behaviors of osteoclasts. Most of these inhibitory effects—except for the increase in pyroptosis—could be reversed by the STAT3 activator colivelin. Therefore, we propose that niclosamide influences osteoclasts primarily through the STAT3 pathway. The mechanisms underlying its effect on pyroptosis warrant further investigation.

Osteoclasts are derived from the monocyte/macrophage hematopoietic lineage. Two key extracellular factors, M-CSF and RANKL, are essential for inducing the differentiation of OCPs. Their combined actions upregulate the transcription factor NFATc1, thereby driving osteoclast differentiation.^3^ NFATc1 activation promotes the transcription of osteoclast-specific genes, such as TRAP, CTSK, and matrix metalloproteinase-9 (MMP-9), which play critical roles in osteoclast formation and the bone-resorptive function of mature osteoclasts.^38^ Microphthalmia-associated transcription factor shares many features with NFATc1, including overlapping transcriptional targets,^39–41^ and both are key regulators of osteoclast differentiation and fusion.^42^^,^^43^ Studies have shown that during osteoclast differentiation, MITF, Spi-1 proto-oncogene (PU.1), and activated NFATc1 cooperatively activate the promoters of TRAP, CTSK, and osteoclast-associated receptor (OSCAR), thereby increasing the transcription of key genes in osteoclasts.^39^^,^^44^ Moreover, the negative regulatory axis NFATc1–Blimp-1–Bcl-6 also modulates osteoclast differentiation. Normally, NFATc1 promotes Blimp-1 expression, which in turn suppresses Bcl-6 expression. An increase in Bcl-6 expression can act as a feedback loop to inhibit the expression of osteoclast-related genes, such as dendrocyte expressed seven transmembrane protein (DC-STAMP), NFATc1, and CTSK, resulting in the formation of a negative regulatory circuit.^45^^,^^46^ Together, these mechanisms maintain normal osteoclast differentiation. In our study, we found that niclosamide suppressed the expression of NFATc1, MITF, Blimp-1, and their downstream osteoclast-specific gene TRAP while upregulating the expression of the negative regulator Bcl-6, thereby inhibiting osteoclast differentiation. These effects appear to be closely linked to STAT3 signaling.

Bone resorption by osteoclasts is a complex and dynamic process in which osteoclasts attach tightly to the bone surface via an actin ring, resulting in the formation of resorption lacuna between the cell and the bone matrix. The basolateral membrane facing the bone develops an elaborate folded structure known as the ruffled border, through which matrix-degrading enzymes, hydrogen ions (H^+^), and chloride ions (Cl^−^) are secreted to acidify the local microenvironment, thereby promoting the dissolution of inorganic minerals and providing the acidic conditions required for key resorptive enzymes such as CTSK to degrade the organic bone matrix and execute resorptive functions.^9^^,^^47^ The acidification of resorption lacuna (pH: 4.5) is achieved by the concerted actions of V-ATPase and CLCN7, both of which are densely localized at the ruffled border.^11^^,^^48^ V-ATPase is a large multi-subunit complex composed of at least 14 subunits organized into V1 and V0 domains and situated on the ruffled border membrane to mediate extracellular acidification during bone resorption,^9^ whereas CLCN7 encodes Cl^−^/H^+^ exchanger chloride channel 7 (ClC-7), which is typically localized to lysosomal compartments and the ruffled membrane of resorbing osteoclasts, with mutations in CLCN7 impairing osteoclast acid secretion and leading to osteopetrosis.^11^ In the present study, pH measurements indicated that niclosamide disrupted normal osteoclast acidification and reduced resorption pit areas on bone slices, further demonstrating an impaired bone-resorptive function, whereas in vivo, niclosamide partially ameliorated bone loss in ovariectomized mice. Moreover, the expression of key genes involved in acid secretion, including CTSK, V-ATPase, and CLCN7, was significantly suppressed by niclosamide, and the reactivation of STAT3 restored the acid-secreting capacity of osteoclasts, highlighting the dependence of this functional axis on STAT3 signaling.

Osteoporosis has been shown to be closely associated with increased recruitment of OCPs, suggesting that modulating the chemotaxis of OCPs to bone remodeling sites could reverse bone loss. The primary chemotactic mediators of BM-derived OCPs include CXCL12 and S1P.^19^ Studies have shown that S1PR1 and S1PR2 are preferentially expressed on OCPs. Under physiological conditions, relatively high concentrations of CXCL12 and low levels of S1P are detected in the BM, a pattern opposite to that in plasma. In pathological states, this balance shifts toward S1P dominance, leading to the S1PR-driven chemotactic attraction of OCPs toward the circulation, where S1P levels are elevated. In high-S1P environments, S1PR1 is internalized, while S1PR2 becomes dominant. Consequently, OCPs migrate back to the BM via S1PR2-mediated chemorepulsion. A study of S1PR2-deficient mice has shown increased bone mass and trabecular density due to reduced osteoclast attachment and subsequent resorption.^18^ Clinical evidence further indicates that elevated circulating S1P levels may serve as an independent risk factor for osteoporotic fractures. In our study, we observed that niclosamide treatment reduced S1P concentrations both in the culture supernatants during osteoclast differentiation and in the serum of mice in vivo. Concurrently, the expression of key chemotaxis-related genes was also suppressed.

Pyroptosis is a form of programmed cell death that is distinct from apoptosis and is characterized by inflammasome activation, resulting in cellular swelling, plasma membrane rupture, and the release of intracellular contents that trigger a robust inflammatory response.^23^ Morphologically, compared with necrotic cells, pyroptotic cells exhibit intermediate features between apoptosis and necrosis, resulting in less swelling and the generation of numerous membrane-bound vesicles (pyroptotic bodies) prior to membrane rupture. In our study, mature osteoclasts treated with niclosamide for 24 h died, and SEM revealed morphological changes consistent with pyroptosis. Consequently, we examined the expression levels of pyroptosis-related genes, including NLRP3, ASC, and GSDMD.^24^ The results showed that niclosamide upregulated the expression of these genes, leading to pyroptosis in mature osteoclasts. Notably, these effects were not reversed by colivelin, suggesting that niclosamide-induced pyroptosis may operate independently of the STAT3 pathway.

Several limitations of this study should be acknowledged when interpreting the findings. First, in the osteoblast–osteoclast co-culture system, we evaluated only the effects of niclosamide on osteoclast differentiation and bone-resorbing activity, without examining the expression of osteoblast marker genes (eg, Runx2, Bglap, and Alpl) or performing bone histomorphometric analyses of osteoblast number and bone formation parameters in the mouse model. Given that niclosamide has also been reported to inhibit the Wnt/β-catenin pathway—a critical regulatory axis for osteoblast differentiation—the present data cannot exclude a direct effect of the drug on osteoblasts, nor can they fully capture its net impact on bone remodeling balance. Second, the primary BMM culture system may contain trace amounts of mesenchymal stromal cells or other BM-derived cells, which could indirectly influence osteoclast differentiation through paracrine mechanisms; thus, a non-cell-autonomous contribution cannot be completely ruled out. In addition, previous studies have suggested that the JAK/STAT3 pathway is not absolutely required for osteoclast differentiation and function, and niclosamide itself is a multi-target agent. Therefore, the present findings can only suggest that STAT3 is an important contributor to the observed effects, rather than defining it as the sole dependent mechanism. Third, with respect to the modulation of osteoclast chemotaxis by niclosamide (see “Effects of niclosamide on osteoclast chemotaxis”), our observations currently rely solely on mRNA expression profiling of chemokines and their receptors; direct functional evidence from Transwell migration or chemotaxis assays is lacking. Consequently, the precise effect of niclosamide on the chemotactic activity of osteoclasts remains to be defined. These limitations represent important directions for future investigation, and further studies using conditional gene KO or purified cell populations are warranted to clarify the cell-autonomous and pathway-specific effects of niclosamide.

Our findings indicate that niclosamide likely influences osteoclast differentiation, fusion, acid secretion, and chemotaxis via the STAT3 pathway, thereby inhibiting osteoclast-mediated bone resorption and partially reversing bone loss in ovariectomized mice. However, the animal model we established replicates only the pathogenesis of postmenopausal OP in females. The initiation and progression of primary OP still require further investigation. Additionally, we observed that niclosamide promotes pyroptosis in mature osteoclasts; however, this effect appears to be independent of STAT3 signaling, and its underlying molecular mechanism warrants further investigation. In summary, the results of this study demonstrate that niclosamide modulates multiple biological functions of osteoclasts and exerts bone-protective effects, suggesting a novel therapeutic strategy for the clinical management of OP.

## Supplementary Material

Supplementary_Material_for_Publication_ziag169

## Data Availability

The datasets generated and/or analyzed during the current study are available from the corresponding author on reasonable request.

## References

[1] Goltzman D . Discoveries, drugs and skeletal disorders. Nat Rev Drug Discov. 2002;1(10):784–796. 10.1038/nrd916 12360256

[2] Hadjidakis DJ, Androulakis II. Bone remodeling. Ann N Y Acad Sci. 2006;1092(1):385–396. 10.1196/annals.1365.035 17308163

[3] Boyle WJ, Simonet WS, Lacey DL. Osteoclast differentiation and activation. Nature. 2003;423(6937):337–342. 10.1038/nature01658 12748652

[4] Feng X, Mcdonald JM. Disorders of bone remodeling. Annu Rev Pathol. 2011;6(1):121–145. 10.1146/annurev-pathol-011110-130203 PMC3571087 20936937

[5] Väänänen K . Mechanism of osteoclast mediated bone resorption--rationale for the design of new therapeutics. Adv Drug Deliv Rev. 2005;57(7):959–971. 10.1016/j.addr.2004.12.018 15876398

[6] Galibert L, Tometsko ME, Anderson DM, Cosman D, Dougall WC. The involvement of multiple tumor necrosis factor receptor (TNFR)-associated factors in the signaling mechanisms of receptor activator of NF-kappaB, a member of the TNFR superfamily. J Biol Chem. 1998;273(51):34120–34127. 10.1074/jbc.273.51.34120 9852070

[7] Wong BR, Josien R, Lee SY, Vologodskaia M, Steinman RM, Choi Y. The TRAF family of signal transducers mediates NF-kappaB activation by the TRANCE receptor. J Biol Chem. 1998;273(43):28355–28359. 10.1074/jbc.273.43.28355 9774460

[8] Feng X . RANKing intracellular signaling in osteoclasts. IUBMB Life. 2005;57(6):389–395. 10.1080/15216540500137669 16012047

[9] Qin A, Cheng TS, Pavlos NJ, Lin Z, Dai KR, Zheng MH. V-ATPases in osteoclasts: structure, function and potential inhibitors of bone resorption. Int J Biochem Cell Biol. 2012;44(9):1422–1435. 10.1016/j.biocel.2012.05.014 22652318

[10] Maeda H, Kowada T, Kikuta J, et al. Real-time intravital imaging of pH variation associated with osteoclast activity. Nat Chem Biol. 2016;12(8):579–585. 10.1038/nchembio.2096 27272564

[11] Kornak U, Kasper D, Bösl MR, et al. Loss of the ClC-7 chloride channel leads to osteopetrosis in mice and man. Cell. 2001;104(2):205–215. 10.1016/S0092-8674(01)00206-9 11207362

[12] Broxmeyer HE, Kohli L, Kim CH, et al. Stromal cell-derived factor-1/CXCL12 directly enhances survival/antiapoptosis of myeloid progenitor cells through CXCR4 and G(alpha)i proteins and enhances engraftment of competitive, repopulating stem cells. J Leukoc Biol. 2003;73(5):630–638. 10.1189/jlb.1002495 12714578

[13] Broxmeyer HE . Chemokines in hematopoiesis. Curr Opin Hematol. 2008;15(1):49–58. 10.1097/MOH.0b013e3282f29012 18043246

[14] Proia RL, Hla T. Emerging biology of sphingosine-1-phosphate: its role in pathogenesis and therapy. J Clin Invest. 2015;125(4):1379–1387. 10.1172/JCI76369 PMC4409021 25831442

[15] Keller J, Catala-Lehnen P, Huebner AK, et al. Calcitonin controls bone formation by inhibiting the release of sphingosine 1-phosphate from osteoclasts. Nat Commun. 2014;5(1):5215. 10.1038/ncomms6215 PMC4205484 25333900

[16] Ishii M, Egen JG, Klauschen F, et al. Sphingosine-1-phosphate mobilizes osteoclast precursors and regulates bone homeostasis. Nature. 2009;458(7237):524–528. 10.1038/nature07713 PMC2785034 19204730

[17] Wang C, Mao J, Redfield S, Mo Y, Lage JM, Zhou X. Systemic distribution, subcellular localization and differential expression of sphingosine-1-phosphate receptors in benign and malignant human tissues. Exp Mol Pathol. 2014;97(2):259–265. 10.1016/j.yexmp.2014.07.013 PMC4177454 25084322

[18] Ishii M, Kikuta J, Shimazu Y, Meier-Schellersheim M, Germain RN. Chemorepulsion by blood S1P regulates osteoclast precursor mobilization and bone remodeling in vivo. J Exp Med. 2010;207(13):2793–2798. 10.1084/jem.20101474 PMC3005230 21135136

[19] Golan K, Kollet O, Lapidot T. Dynamic cross talk between S1P and CXCL12 regulates hematopoietic stem cells migration, development and bone remodeling. Pharmaceuticals (Basel). 2013;6(9):1145–1169. 10.3390/ph6091145 PMC3818832 24276423

[20] Ratajczak MZ, Lee H, Wysoczynski M, et al. Novel insight into stem cell mobilization-plasma sphingosine-1-phosphate is a major chemoattractant that directs the egress of hematopoietic stem progenitor cells from the bone marrow and its level in peripheral blood increases during mobilization due to activation of complement cascade/membrane attack complex. Leukemia. 2010;24(5):976–985. 10.1038/leu.2010.53 PMC2946378 20357827

[21] Ryu J, Kim HJ, Chang EJ, Huang H, Banno Y, Kim HH. Sphingosine 1-phosphate as a regulator of osteoclast differentiation and osteoclast-osteoblast coupling. EMBO J. 2006;25(24):5840–5851. 10.1038/sj.emboj.7601430 PMC1698879 17124500

[22] Zhang Y, Chen X, Gueydan C, Han J. Plasma membrane changes during programmed cell deaths. Cell Res. 2018;28(1):9–21. 10.1038/cr.2017.133 PMC5752838 29076500

[23] Liston A, Masters SL. Homeostasis-altering molecular processes as mechanisms of inflammasome activation. Nat Rev Immunol. 2017;17(3):208–214. 10.1038/nri.2016.151 28163301

[24] He WT, Wan H, Hu L, et al. Gasdermin D is an executor of pyroptosis and required for interleukin-1β secretion. Cell Res. 2015;25(12):1285–1298. 10.1038/cr.2015.139 PMC4670995 26611636

[25] Redondo-Castro E, Faust D, Fox S, et al. Development of a characterised tool kit for the interrogation of NLRP3 inflammasome-dependent responses. Sci Rep. 2018;8(1):5667. 10.1038/s41598-018-24029-3 PMC5884858 29618797

[26] Tao H, Zhang Y, Zeng X, Shulman GI, Jin S. Niclosamide ethanolamine-induced mild mitochondrial uncoupling improves diabetic symptoms in mice. Nat Med. 2014;20(11):1263–1269. 10.1038/nm.3699 PMC4299950 25282357

[27] Li Y, Li PK, Roberts MJ, Arend RC, Samant RS, Buchsbaum DJ. Multi-targeted therapy of cancer by niclosamide: a new application for an old drug. Cancer Lett. 2014;349(1):8–14. 10.1016/j.canlet.2014.04.003 PMC4166407 24732808

[28] Lu W, Lin C, Roberts MJ, Waud WR, Piazza GA, Li Y. Niclosamide suppresses cancer cell growth by inducing Wnt co-receptor LRP6 degradation and inhibiting the Wnt/β-catenin pathway. PLoS One. 2011;6(12):e29290. 10.1371/journal.pone.0029290 PMC3241710 22195040

[29] You S, Li R, Park D, et al. Disruption of STAT3 by niclosamide reverses radioresistance of human lung cancer. Mol Cancer Ther. 2014;13(3):606–616. 10.1158/1535-7163.MCT-13-0608 PMC3964811 24362463

[30] Jin Y, Lu Z, Ding K, et al. Antineoplastic mechanisms of niclosamide in acute myelogenous leukemia stem cells: inactivation of the NF-kappaB pathway and generation of reactive oxygen species. Cancer Res. 2010;70(6):2516–2527. 10.1158/0008-5472.CAN-09-3950 20215516

[31] Wang AM, Ku HH, Liang YC, Chen YC, Hwu YM, Yeh TS. The autonomous notch signal pathway is activated by baicalin and baicalein but is suppressed by niclosamide in K562 cells. J Cell Biochem. 2009;106(4):682–692. 10.1002/jcb.22065 PMC7166476 19160421

[32] Osada T, Chen M, Yang XY, et al. Antihelminth compound niclosamide downregulates Wnt signaling and elicits antitumor responses in tumors with activating APC mutations. Cancer Res. 2011;71(12):4172–4182. 10.1158/0008-5472.CAN-10-3978 PMC3117125 21531761

[33] Yang Y, Chung MR, Zhou S, et al. STAT3 controls osteoclast differentiation and bone homeostasis by regulating NFATc1 transcription. J Biol Chem. 2019;294(42):15395–15407. 10.1074/jbc.RA119.010139 PMC6802509 31462535

[34] Shen G, Ren H, Shang Q, et al. Foxf1 knockdown promotes BMSC osteogenesis in part by activating the Wnt/β-catenin signalling pathway and prevents ovariectomy-induced bone loss. EBioMedicine. 2020;52:102626. 10.1016/j.ebiom.2020.102626 PMC6992955 31981979

[35] Jiao Y, Chen C, Hu X, et al. Niclosamide and its derivative DK-520 inhibit RANKL-induced osteoclastogenesis. FEBS Open Bio. 2020;10(8):1685–1697. 10.1002/2211-5463.12921 PMC7396435 32602250

[36] Özpak Akkuş Ö, Atalay B. Post-menopausal osteoporosis: do body composition, nutritional habits, and physical activity affect bone mineral density? Nutr Hosp. 2020;37(5):977–983. 10.20960/nh.03214 32960644

[37] Post TM, Cremers SC, Kerbusch T, Danhof M. Bone physiology, disease and treatment: towards disease system analysis in osteoporosis. Clin Pharmacokinet. 2010;49(2):89–118. 10.2165/11318150-000000000-00000 20067335

[38] Takayanagi H, Kim S, Koga T, et al. Induction and activation of the transcription factor NFATc1 (NFAT2) integrate RANKL signaling in terminal differentiation of osteoclasts. Dev Cell. 2002;3(6):889–901. 10.1016/S1534-5807(02)00369-6 12479813

[39] Sharma SM, Bronisz A, Hu R, et al. MITF and PU.1 recruit p38 MAPK and NFATc1 to target genes during osteoclast differentiation. J Biol Chem. 2007;282(21):15921–15929. 10.1074/jbc.M609723200 17403683

[40] So H, Rho J, Jeong D, et al. Microphthalmia transcription factor and PU.1 synergistically induce the leukocyte receptor osteoclast-associated receptor gene expression. J Biol Chem. 2003;278(26):24209–24216. 10.1074/jbc.M302940200 12695521

[41] Kim K, Kim JH, Lee J, et al. Nuclear factor of activated T cells c1 induces osteoclast-associated receptor gene expression during tumor necrosis factor-related activation-induced cytokine-mediated osteoclastogenesis. J Biol Chem. 2005;280(42):35209–35216. 10.1074/jbc.M505815200 16109714

[42] Kim K, Lee SH, Ha Kim J, Choi Y, Kim N. NFATc1 induces osteoclast fusion via up-regulation of Atp6v0d2 and the dendritic cell-specific transmembrane protein (DC-STAMP). Mol Endocrinol. 2008;22(1):176–185. 10.1210/me.2007-0237 PMC2725751 17885208

[43] Thesingh CW, Scherft JP. Fusion disability of embryonic osteoclast precursor cells and macrophages in the microphthalmic osteopetrotic mouse. Bone. 1985;6(1):43–52. 10.1016/8756-3282(85)90406-5 3994857

[44] Luchin A, Suchting S, Merson T, et al. Genetic and physical interactions between Microphthalmia transcription factor and PU.1 are necessary for osteoclast gene expression and differentiation. J Biol Chem. 2001;276(39):36703–36710. 10.1074/jbc.M106418200 11481336

[45] Nishikawa K, Nakashima T, Hayashi M, et al. Blimp1-mediated repression of negative regulators is required for osteoclast differentiation. Proc Natl Acad Sci USA. 2010; 107(7):3117–3122. 10.1073/pnas.0912779107 PMC2840297 20133620

[46] Miyamoto T . Regulators of osteoclast differentiation and cell-cell fusion. Keio J Med. 2011;60(4):101–105. 10.2302/kjm.60.101 22200633

[47] Kartner N, Manolson MF. Novel techniques in the development of osteoporosis drug therapy: the osteoclast ruffled-border vacuolar H(+)-ATPase as an emerging target. Expert Opin Drug Discov. 2014;9(5):505–522. 10.1517/17460441.2014.902155 24749538

[48] Blair HC, Teitelbaum SL, Ghiselli R, Gluck S. Osteoclastic bone resorption by a polarized vacuolar proton pump. Science. 1989;245(4920):855–857. 10.1126/science.2528207 2528207

