# Supplementary material for: Niclosamide suppresses osteoclastogenesis and bone resorption: involvement of Signal Transducer And Activator Of Transcription 3 signaling
Source: JBMR Plus. 2026 Oct 19;10(12):ziag169. doi: 10.1093/jbmrpl/ziag169 (PMC13744740; doi:10.1093/jbmrpl/ziag169)
Supplement: Supplementary file 1 [file supplementary_material_for_publication_ziag169.docx]

**Supplementary Table 1. Primer sequences**

| Gene Name | Forward (5’–3’) | Reverse (5’–3’) |
| --- | --- | --- |
| GAPDH | AGGTCGGTGTGAACGGATTTG | TGTAGACCATGTAGTTGAGGTCA |
| NFATC1 | CAACACCAAAGTCCTGGAGATCC | ATGTGAACTCGGAAGACCAGCCT |
| Mitf | TATGAACACGCACTCTCGAGC | CAAGGCCGGATCCATCAAGC |
| Blimp1 | AAGAGGTTATTGGCGTGGTAAG | TAGACTTCACCGATGAGGGGT |
| Bcl6 | CCGGCACGCTAGTGATGTT | TGTCTTATGGGCTCTAAACTGCT |
| Trap5 | TACTTCACTGGAGTGCACGATG | GAAGCGCAAACGGTAGTAAGG |
| V-ATPase | CAGAGCTGTACTTCAATGTGGAC | AGGTCTCACACTGCACTAGGT |
| CLCN7 | AACAACGTGGAGCTGGATGAC | GGAAGAGCTGATTCTCGCTGT |
| CTSK | CAGTAGCCACGCTTCCTATCC | GAGACAGAGCAAAGCTCACCA |
| SPHK1 | TGAGGTGGTGAATGGGCTAATGGA | AACAGCAGTGTGCAGTTGATGAGC |
| SPNS2 | GGCATCTTCTTCTGGTCTGCTG | TGCTTCACGCTGGAACCTGTGA |
| S1PR1 | TCTCGGACCTATTAGCAGGCGT | AGCATGGTGATGTAGCGCTCA |
| S1PR2 | CTCATTGCAGTGGCCAGGAACA | GAGAGTAAGGTGTTGGCCACGA |
| CXCL12 | CAGAGCCAACGTCAAGCATCT | TCCAGGTACTCTTGGATCCACT |
| CXCR4 | GAAGCTAAGGAGCATGACGGA | GATGAGAACGCTGCTGTAGAGG |
| NLRP3 | GACTGGCAAAAGGCTGTG | TTGGATCAGCCTGAAGGCC |
| Caspase 1 | GCTCTGCGGTGTAGAAAAG | ATAGGTCCCGTGCCTTG |
| IL-1β | AGTTGACGGACCCCAAA | ACAGAGGATGGGCTCTTCT |
| GSDMD | AGTGCCTCCATGAATGTGTG | TGCAGGAATGGTCACCATCT |
| ASC | CTGCAGATGGACGCCATAGA | TTCAGTCAGCACACTGCCATGC |

**Supplementary Figure 1.** (a) Osteoclast monocultures treated with niclosamide at the early differentiation stage were maintained for an extended period, with TRAP staining performed on day 5 after RANKL treatment in the control and rescue groups and on day 6 in the niclosamide group (scale bars: 400 μm, 100 μm, and 50 μm) (b) Quantification of the mature osteoclast area in each group. (c) Osteoclast–osteoblast coculture system. (d) and (e) TRAP staining of osteoclast monocultures and cocultures, respectively, at 0, 1, 3, 6, 12, and 24 hours after niclosamide treatment was administered after differentiation/fusion (scale bars for e: 100 μm and 50 μm). (f) Representative dual‑energy X‑ray absorptiometry (DXA) scan of mouse bone mineral density (BMD). (g) and (h) Whole‑body and spinal BMD measurements by DXA to validate the ovariectomy‑induced osteoporosis model (scale bar in g: 1 cm). (i) and (j) Whole‑body and spinal BMD among treatment groups after the drug intervention. (k) Summary of the changes in whole‑body and spinal BMD in the niclosamide group before and after the intervention (****P* < 0.001, ***P* < 0.01, and **P* < 0.05).
